# BRG1 Activates Proliferation and Transcription of Cell Cycle-Dependent Genes in Breast Cancer Cells

**DOI:** 10.3390/cancers12020349

**Published:** 2020-02-04

**Authors:** Maciej Sobczak, Julita Pietrzak, Tomasz Płoszaj, Agnieszka Robaszkiewicz

**Affiliations:** 1 Department of General Biophysics, Institute of Biophysics, Faculty of Biology and Environmental Protection, University of Lodz, Pomorska 141/143, 90-236 Lodz, Poland; maciej.sobczak@unilodz.eu (M.S.); julita.pietrzak@unilodz.eu (J.P.); 2 Department of Clinical and Laboratory Genetics, Medical University of Lodz, Pomorska 251, 92-213 Lodz, Poland; tomasz.ploszaj@umed.lodz.pl

**Keywords:** BRG1 (Brahma-Related Gene 1), EP300 (E1A Binding Protein P300), gene transcription, breast cancer epigenetics

## Abstract

Cancer malignancy is usually characterized by unlimited self-renewal. In some types of advanced tumors that are rapidly dividing, gene expression profiles depict elevations in pro-proliferative genes accompanied by coordinately elevated transcription of factors responsible for removal of DNA lesions. In our studies, fast proliferating breast cancer cell lines (MDA-MB-231 and MCF7), BRG1, a component of the SWI/SNF complex, emerges as an activator of functionally-linked genes responsible for activities such as mitotic cell divisions and DNA repair. Products of at least some of them are considerably overrepresented in breast cancer cells and BRG1 facilitates growth of MCF7 and MDA-MB-231 cell lines. BRG1 occurs at the promoters of genes such as *CDK4*, *LIG1*, and *NEIL3*, which are transcriptionally controlled by cell cycle progression and highly acetylated by EP300 in proliferating cells. As previously documented, in dividing cells BRG1 directly activates gene transcription by evicting EP300 modified nucleosomes from the promoters and, thereby, relaxing chromatin. However, the deficiency of BRG1 or EP300 activity for 48 h leads to cell growth arrest and to chromatin compaction, but also to the assembly of RB1/HDAC1/EZH2 complexes at the studied cell cycle-dependent gene promoters. Epigenetic changes include histone deacetylation and accumulation of H3K27me trimethylation, both known to repress transcription. Cell cycle arrest in G1 by inhibition of CDK4/6 phenocopies the effect of the long-term BRG1 inhibition on the chromatin structure. These results suggest that BRG1 may control gene transcription also by promoting expression of genes responsible for cell cycle progression in the studied breast cancer cells. In the current study, we show that BRG1 binding occurs at the promoters of functionally linked genes in proliferating breast cancer cells, revealing a new mechanism by which BRG1 defines gene transcription.

## 1. Introduction

Genomic instability and deactivation of DNA repair genes are often associated with tumor-prone phenotypes and necessary for acquisition of tumor initiating mutations. However, from a certain moment of malignancy, the genetic stabilization and maintenance of genome integrity might be required in order to invade tissues and give rise to distant metastases, but the increased ability to repair DNA damage seems to depend on tumor type [1]. Transcription control and the epigenetic landscape that confers high expression of DNA repair genes in some types of malignant cells remain poorly explored. Relatively recent findings indicate that uncontrolled growth and cell division dominate tumor transcriptional programs, and that genes relevant to the removal of DNA damage correlate with the proliferative status of the tumors [2]. Once again, direct molecular and mechanistical mutual interdependence has not been forthcoming. 

Our recently published results provide evidence that transcription of some of DNA repair genes, such as *PARP1*, *BRCA1*, and *XRCC1*, is controlled by SWI/SNF in a cell cycle-dependent manner [3]. In our model of monocyte-to-macrophage differentiation promoters of DNA repair genes, characterized by the presence of CpG island and E2F motifs, were enriched in SWI/SNF-EP300-HDAC1 complexes. Macrophage differentiation that re-entered cell proliferation switched between BRM to BRG1 and HDAC1 to EP300 activity, thus it turned on gene transcription. In brief, in growth arrested monocytes EP300 remained inactive, while HDAC1 deacetylated gene promoters thereby preventing nucleosome eviction by BRM and leading to gene silencing. In proliferating macrophages, EP300 modified nucleosomes, while BRG1 served as acetylation reader and extruded acetylated histones. However, the interdependence of cell proliferation status, EP300 vs. HDAC1 and BRM vs. BRG1 activity remained unexplored.

With respect to other genes and cell types, the role of BRG1 remains ambiguous and a molecular mechanism that turns this enzyme into a gene activator or repressor has not been found. It might be linked with co-operating factors, nucleosome modifications or histone variants, which appear at a given place and time at chromatin [4,5,6]. Data collected for human monocytes and macrophages provided the first mechanistic insight into proliferation control of gene expression that was mediated by SWI/SNF-EP300-HDAC1 complexes assembled at E2F binding sites. E2F motifs emerged as genetic signatures of the occurrence of BRG1 in the genome. These findings prompted us to test if similar interdependence between BRG1 distribution in genome and gene overexpression occurs in cancer cells, since, in terms of cell cycle status, development of malignancies resembles differentiation of human proliferating macrophages from growth arrested monocytes. 

E2F motifs mark proliferation-sensitive gene promoters, which respond to growth arrest by recruiting retinoblastoma-based repressive complexes comprising (depending on the biological circumstances) RB1, RBL1, and RBL2 as its basic components. These proteins co-occur with numerous chromatin writers and erasers, including HDACs, PRC2, DNMT1, SUV39H1, or HP1, all capable of suppressing gene transcription [7,8,9]. Since in the current study we observed that BRG1 promotes cell cycle progression in the breast cancer cells and that some of DNA repair genes are controlled by the cell proliferation status, we took into consideration a possible functional cross-talk between BRG1 activity, cell divisions and formation of RB1-based repressive complexes. A relatively older report noted BRG1 and RB1 co-operating physically and functionally in the human carcinoma cell line SW13 [10]; however, both proteins contributed to growth arrest. In the other study, BRG1-containing complexes were shown to control cellular proliferation by upregulation of p21 and, thereby, hypophosphorylation of pRB and repression of E2F target genes such as *CDK2*, *CCNE*, and *CCND* [11].

In the current study, we reveal additional complexity to how BRG1 can modulate E2F-dependent transcription. In our model, BRG1 occurs at E2F/CpG-positive, highly acetylated promoters of genes that are overexpressed in breast primary tumor, and two selected highly invasive breast cancer cell lines: MCF7 and MDA-MB-231. Among BRG1-enriched promoters we found genes encoding factors responsible for cancer cell proliferation and resistance to DNA damage. BRG1 dually activates their transcription: (a) directly by acting at the chromatin level and evicting acetylated nucleosomes from their promoters, and (b) indirectly by potentiating cell proliferation and preventing assembly of RB1-HDAC1-PRC2 repressive complexes at the gene promoters. The E2F binding motif at the promoters of some genes, which are functionally linked to cell proliferation and DNA repair in the studied breast cancer cells, allow BRG1-EP300 complexes to provide a common mechanism of gene transcription control.

## 2. Results

### 2.1. E2F/CpG Motifs at the Acetylated Gene Promoters Mark BRG1 Distribution in Genome of Breast Cancer Cells

**Figure 1:**
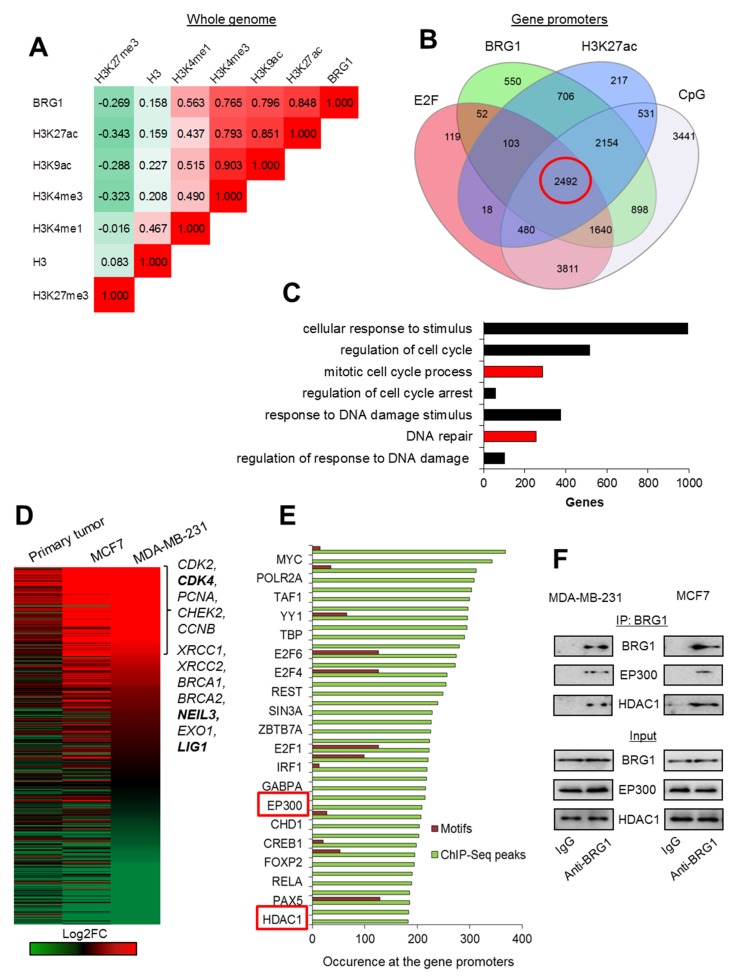
BRG1 occurs at the acetylated promoters of some highly transcribed genes, which control proliferation and DNA repair in breast cancer cells. (**A**) BRG1 co-distribution with histone H3 density and histone modifications in the genome of MDA-MB-231 is shown as Pearson’s correlation coefficient. (**B**) Occurrence of BRG1 at the acetylated gene promoters characterized by E2F binding site and CpG island has been quantified on a Venn diagram and BRG1/H3K27ac/E2F/CpG promoters are marked in red circle. Green and blue circles represent gene promoters enriched in BRG1 and H3K27ac peaks according to MACS, while grey and red represent promoters featured by the presence of CpG islands according to cpgIslandExt and E2F binding motifs according to cpgIslandExt and wgEncodeRegTfbsClusteredV3, respectively. (**C**) Functional association of BRG1/H3K27ac/E2F/CpG gene promoters (marked in red circle in (**B**) leads to enrichment of intracellular processes that can define cancer physiology. Red bars represent biological processes, which are taken for further analysis in (**D**) and (**E**). (**D**) Analysis of differential gene expression from data derived from RNA-Seq confirms overexpression of genes functionally assigned to the mitotic cell cycle and to responses to stimuli of DNA damage in cancer cell lines versus normal breast cells. Genes marked in bold were taken as examples for further analysis in Figure 2A–D. (**E**) BRG1/H3K27ac/E2F/CpG promoters of genes overexpressed in cancer cells (**D**): Log2FC > 0.5 for at least 2 of the cell types used are characterized by common transcription factors and chromatin remodelers. Green columns correspond to the number of ChIP-Seq peak occurrences at the gene promoters according to UCSC wgEncodeRegTfbsClusteredV3, whereas red columns represent the occurrence of transcription factor binding motifs according to tfbsConsSites. Only every other transcription factor is labeled. (**F**) Immunoprecipitation of BRG1 allows to detect EP300 and HDAC1 by western blot and indicates the physical interaction between SWI/SNF component and the latter two enzymes. Pictures show cropped areas of western blots. Whole images are included in Figure S3.

To test if BRG1 may contribute to transcription regulation of genes in fast proliferating breast cancer cells, we investigated whether this enzyme co-occurs genome-wide with any particular histone mark that is known for its involvement in transcription control. For this analysis, we took publicly available data from ChIP-Seq experiments for BRG1 and selected histone modifications, and calculated Pearson correlation coefficient between their co-distribution in the genome of MDA-MB-231 cells. Genomic occurrence of BRG1 showed it was most strongly correlated with histone acetylation and H3K4me3, which are usually associated with gene promoters and active transcription (Figure 1A). Lack of reciprocity between enzyme and H3, as well as weak negative co-occurrence with H3K27me3, seem to further confirm a previously postulated mechanism, where BRG1 evicted histones from transcriptionally permissive promoters and enabled gene expression. In human macrophages, BRG1/H3K27ac-positive promoters are characterized by binding motif for E2F (indicative of likely gene dependence on cell cycle status) and/or the CpG island [3]. To test whether distribution of BRG1 is associated with similar chromatin and DNA features in proliferating breast cancer cells, MDA-MB-231, we looked for overlapping regions adjacent to TSS (±2 kbp), which are characterized by the occurrence of BRG1, H3K27ac, E2F motifs, and CpG islands. As shown in Figure 1B and Table S1, the great majority of BRG1-rich promoters was simultaneously acetylated and featured by CpG island, while to a lower extent by E2F motif. This analysis also supported the previously postulated mutual interdependence between occurrence of BRG1 and H3K27ac at the gene promoters.

Promoters characterized by all four considered features—BRG1/H3K27ac/E2F/CpG—were associated with genes involved in key cellular processes, among them DNA repair and mitotic divisions (Figure 1C, Table S2). Analysis of differential expression of genes belonging to the two mentioned GO groups showed that substantial part of these genes was overexpressed in primary tumor and 2 cancer cell lines compared to normal cells (Figure 1D). Genes that were suppressed in cancer cells were not further repressed by SWI/SNF inhibition, suggesting that BRG1 association with acetylated promoters is insufficient under certain chromatin circumstances to ensure a high transcription rate. Among the top 50 transcription factors and chromatin remodelers that appear at the promoters of genes overexpressed in cancer cells (Log2FC > 0.4), we identified enzymes EP300 and HDAC1, which we previously showed to create a functional unit with BRG1 at gene promoters in human macrophages (Figure 1E). The presence of TBP and POLR2A suggests that BRG1-based complexes may also contribute to polymerase release or pausing. POLR2A pre-initiation complexes are documented to often feature CpG-driven promoters. Since the overall majority of BRG1/H3K27ac promoters (>85%) carry CpG signature and only approximately half of them the motif for E2F, CpG islands might be considered a predictive hallmark of BRG1-dependent gene transcription. Nevertheless, chromatin-bound or signaling factors that suppress transcription from BRG1/H2K27ac promoters have yet to be identified. 

To check if BRG1 creates a multi-enzyme complex with EP300 and HDAC1 in proliferating cancer cells, we searched for the latter two enzymes in BRG1 co-immunoprecipitates. As shown in Figure 1F, immunoprecipitation of BRG1 confirmed the physical interaction between the studied SWI/SNF component, acetylase and deacetylase. Furthermore, EP300 and BRG1 were also recently documented by us to create a functional and transcription regulating unit with PARP1 in MCF7 and MDA-MB-231 cells, where PARP1 poly-ADP-ribosylated and, thereby, activated EP300 [11]. 

### 2.2. BRG1-EP300 Complexes Drive Transcription of Some Proliferation and DNA Repair Genes

**Figure 2:**
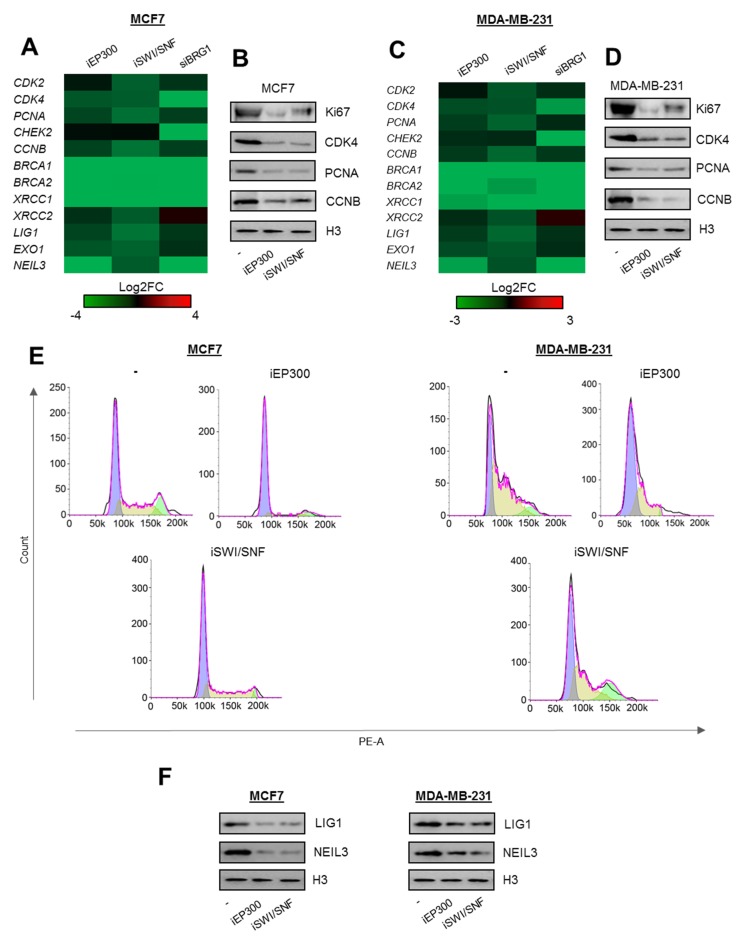
Proliferation and DNA repair-relevant gene promoters are controlled by BRG1 and EP300 activity in breast cancer cells. (**A** and **C**) Long-term (48 h) deficiency of BRG1 and EP300 activity limits transcription of genes encoding components of cell division and DNA repair mechanisms. The data present Log2FC of mRNA level measured by real-time PCR normalized to untreated control (iEP300 and iSWI/SNF) and non-template control (siBRG1). (**B** and **D**) Gene repression results in substantial loss of proliferation markers as seen from western blot, and by cell growth arrest in G1 phase (**E**). (**F**) Inhibition of BRG1 and EP300 similarly decreases the amount of proteins involved in removal of DNA damage. Heatmaps with long-term BRG1 and Ep300 deficiency (**A** and **C**) were prepared from four independent replicates for iEp300, and iSWI/SNF; from 3 for siBRG1. Detailed statistical analysis is presented in supplementary Table S3. Western blots in B, D, and F show representative images from three independent experiments. Pictures show cropped areas of western blots. Whole images are included in Figure S3. Analysis of the cell cycle was carried out twice in two technical replicates for each condition.

To verify the possible contribution of BRG1-EP300 to transcription regulation of selected genes functionally linked to proliferation and removal of DNA damage, we treated MCF7 and MDA-MB-231 cells with inhibitors of EP300 (iEP300–C646–blocks acetylatransferase activity of EP300 and CBP), BRG1 (iSWI/SNF–PFI3 is a bromodomain inhibitor of BRG1 and BRM) and calculated Log2FC of mRNA level quantified by real-time PCR between inhibitor treated and control cells. A high transcription rate of at least some genes, which were found overexpressed in all cancer cell types according to Figure 1D, crucial for cell divisions (*CDK2*, *CDK4*, *PCNA*, *CHEK2*, *CCNB*) and removal of DNA damage (*BRCA1* and *BRCA2*, *XRCC1* and *XRCC2*, *LIG1*, *EXO1*, *NEIL3*—contribute to BER, NER, SSBR, MMR, and HR) [12,13], was simultaneously maintained by BRG1 and EP300 (Figure 2A,C). Inhibition of both enzymes led to substantial suppression of most genes studied in Figure 2A,C. Since the only commercially available inhibitor of SWI/SNF (PFI-3) also blocks the activity of BRM and BRG1, we targeted the latter enzyme with siRNA (Figure S1A–D). *XRCC2* was the only exception and was upregulated in BRG1 deficiency. Although iEP300 causes simultaneous inhibition of EP300 and CBP, the data for siEP300 (Silencer™ Select Pre-Designed siRNA ID:s534247) is missing due to the fact, that substantial EP300 silencing with siRNA induced dramatic cell death. 

Repression of proliferation-relevant genes by the deficiency of BRG1 and EP300 activity led to a substantial reduction in protein proliferation markers (also others than BRG1 targets—Ki67) and to cell cycle arrest in G1 (Figure 2B,D,E). Similarly, DNA repair gene repression was followed by loss of LIG1 and NEIL3 protein upon EP300 and SWI/SNF inhibition (Figure 2F).

### 2.3. Cell Cycle-Dependent Chromatin Composition Controls Transcription of Proliferation and DNA Repair Genes

To study in detail the molecular mechanism of BRG1 contribution to regulation of transcription and possible functional interdependence with the cell cycle progression, we focused on three promoters of genes, which are crucial for cell proliferation—*CDK4* and effective removal of DNA lesions—*LIG1* and *NEIL3*. As shown in Figure 1B and Figure 2A–F), all three genes are transcriptionally controlled by BRG1 and EP300. Their promoters are featured by the presence of BRG1, EP300, CpG island, and E2F motif. Especially, due to the last feature, they are likely to respond to alterations in cell proliferation status and to assemble RB1-based repressive complexes. Therefore, these promoters were chosen to study the considered cross-talk between BRG1 activity, chromatin remodeling, and cell cycle status. Furthermore, CDK4 deficiency caused by BRG1-dependent gene repression, that in cell cultures results in cell growth arrest, can be easily phenocopied by the use of CDK4/6 inhibitors, which equally arrest cells in G1. Thus, from this point the following hypotheses were tested: (a) BRG1/H3K27ac/CpG/E2F promoters of proliferation and DNA repair genes respond to G1 arrest with decreased gene transcription and formation of RB1-based repressive complexes; (b) long-term inhibition of BRG1-EP300 complexes that results in G1 arrest also leads to RB1-based chromatin remodeling at the studied, cell cycle-dependent promoters.

**Figure 3:**
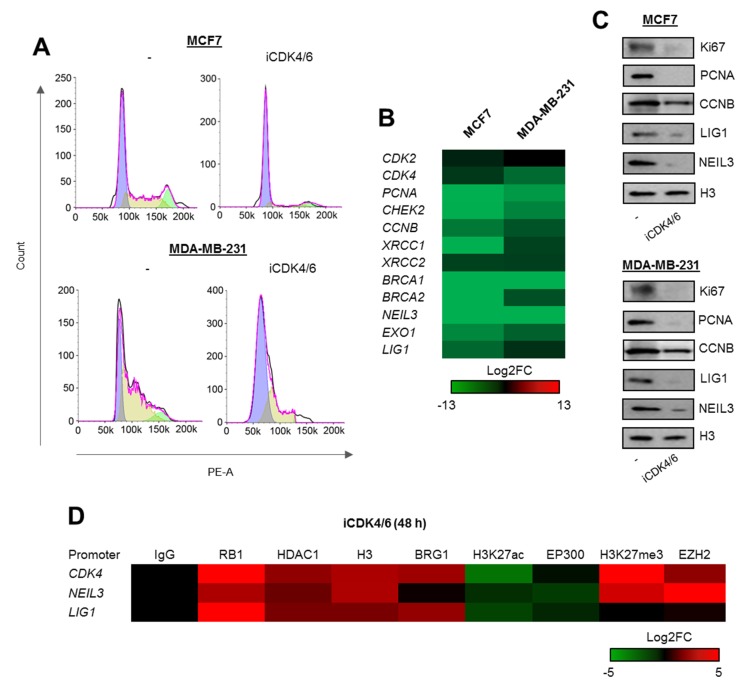
DNA repair genes are transcriptionally controlled by the cell cycle progression. (**A**) Deficiency of CDK4/6 activity results in complete cell cycle blockade in G1. (**B**,**C**) Inhibition of CDK4/6 for 48 h leads to repression of genes that drive cell cycle progression and genome resistance to damage. mRNA ((**B**) shows Log2FC versus untreated) and protein (**C**) was monitored by real-time PCR and western blot in breast cancer cells exposed to iCDK4/6 for 48 h. Pictures in (**C**) show cropped areas of western blots. Whole images are included in Figure S3. (**D**) G1 arrest induces considerable chromatin remodeling and adjustment of protein composition to gene repressive conditions at the promoters of *CDK4*, *NEIL3*, and *LIG1* (ChIP/real-time PCR) 48 h after administration of iCDK4/6 in culture of MCF7 cells. Analysis of the cell cycle progression (**A**) was monitored twice in two technical replicates for each condition, quantification of gene expression in response to iCDK4/6 (**B**) and ChIP/real-time PCR analysis of *CDK4*, *NEIL3* and *LIG1* promoters (**D**) were carried out 4 times, while western blots were repeated 3 times and representative images are shown in (**C**). Detailed statistical data can be found in Table S3.

First, to test hypothesis quoted in (a) we checked whether the arrest of cell cycle progression triggered by the inhibition of cyclin-dependent kinases 4 and 6 (CDK4 and CDK6) that blocks cells in G1 (Figure 3A; the same as iSWI/SNF and iEP300—Figure 2E) suppresses gene expression (Figure 3B), thereby also reducing the protein level (Figure 3C). This led to recruitment of RB1-based repressive complexes to promoters of representative genes: *CDK4*, *LIG1*, and *NEIL3* (the DNA fragment analyzed overlapped the E2F motif within the CpG island) (Figure 3D). All three regulatory regions shared two common features, an increase in H3 density and a decrease in histone acetylation that likely resulted from the switch between EP300 and HDAC1 activity. These phenocopy previously described mechanism that drives proliferation-dependent and BRG1-EP300-HDAC1-mediated chromatin transition from permissive to a compacted structure, where gain of HDAC1 activity in growth arrested cells prevents nucleosome extrusion by BRG1 due to loss of histone acetylation. Unexpectedly, G1 arrest enhances BRG1 association with 2 out of 3 studied gene promoters. However, also inhibition of THP-1 proliferation with mimosine resulted in the massive recruitment of both SWI/SNF ATPases to the promoter of *PARP1* as described in our previous study [14]. As currently we observed such an effect only for two genes, it seems to be promoter or transcription co-factor dependent, but may represent still unknown mechanism of SWI/SNF contribution to repression of cell cycle responsive promoters under certain conditions as documented in Dunaief et al [10]. BRG1 seems to occur and act as activator of transcription by displacing acetylated histones in proliferating cells, but at some gene promoters gets further and strongly enriched upon the growth inhibition. 

Increase in BRG1 association with gene promoters induced by G1 arrest was followed by displacement of EP300, which may (in addition to recruitment and activation of HDAC1) further explain reduced nucleosome acetylation. The assembled repressive complex was supported by polycomb repressive complex 2 (PRC2) at the promoters of *CDK4* and *LIG1*. The enzymatically active subunit of PRC2–EZH2 catalyzed methylation of lysine 27 of histone H3, that is known to cause gene suppression. Of note, PRC2 was found only at two of these gene promoters, and did not correlate with enrichment or extrusion of other possible components of the repressive complex. This may indicate that PRC2 targets chromatin in a promoter rather than in a RB1-dependent manner, and that the presence of RB1 is a prerequisite, but not essential, for PRC2 contribution to gene silencing.

### 2.4. BRG1 Couples Cell Divisions with Transcription of DNA Repair Genes 

**Figure 4:**
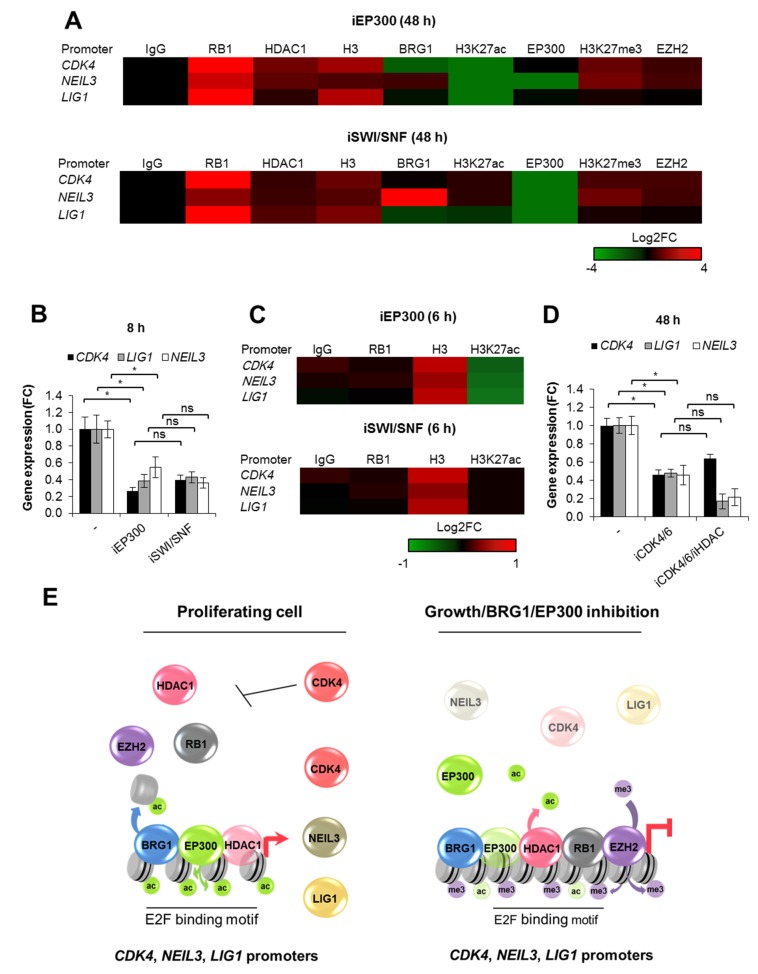
BRG1-dependent cell cycle progression defines chromatin composition at the promoters of DNA repair genes. (**A**) Long-term (48 h) deficiency of BRG1 and EP300 activity induces substantial changes in the chromatin structure, and recruitment of cell cycle-dependent repressors to the promoters of genes involved in cell proliferation and response to DNA damage (ChIP/real-time PCR). (**B**) Short-term (8 h) inhibition of BRG1, EP300, but not CDK4/6, results in transcription restraint (nascent RNA quantification by Click-iT chemistry), and (**C**) immediate chromatin closure without RB1 association with the studied gene promoters (ChIP/real-time PCR) of MCF7 cells. (**D**) Gene transcription (mRNA level; real-time PCR) was measured 48 h after MCF7 cell treatment with iCDK4/6. iHDAC was added 24 h prior to RNA isolation to restore chromatin acetylation. (**E**) Graphic representation of immediate and late chromatin response to cell growth arrest, BRG1 and EP300 inhibition. In the presented model, BRG1 and EP300 stimulate cell proliferation and, therefore, activate transcription of genes, which are transcriptionally driven by cell cycle progression. Data presented in figures (**A**–**D**) are presented as mean of four independent replicates (*n* = 4). Detailed statistical data can be found in Table S3.

To search for a molecular mechanism that links BRG1/EP300 activity with cell cycle-dependent gene transcription, we analyzed the impact of BRG1 and EP300 on chromatin structure at the chosen gene promoters (Figure 4A). Long-term inhibition of both EP300 and SWI/SNF shown to arrest proliferating cells in G1 (Figure 2E) results in substantial chromatin remodeling, with recruitment of RB1, HDAC1, and an increase in nucleosome density. This phenomenon phenocopied the effect of iCDK4/6 inhibition on promoter composition (Figure 3D) and provides evidence that repression of genes, which potentiate cell division, leads to growth arrest-dependent modification of chromatin architecture. As expected, iEP300 reduced acetylation of histones, whereas iSWI/SNF prevented displacement of acetylated nucleosomes, thereby confirming a direct action of BRG1 associated to gene promoters. As for EP300 and BRG1, their occurrence seemed to be controlled by the type of enzyme inhibited (cell treatment with iBRG1 or iEP300) and in a promoter-dependent fashion, but the tendency for EP300 to be displaced from chromatin regardless of the inducer of cell growth arrest was also noted. Consistently to the effect of iCDK4/6, however, EZH2 was not enriched at the *LIG1* promoter, whereas it faintly methylated lysine residues at the promoters of *CDK4* and *NEIL3*.

To confirm that long-term effect of deficiency of BRG1 activity on gene transcription is mediated by BRG1 gene targets which drive cancer cell proliferation (e.g., *CDK4*, *PCNA*, *CCNB*), we checked their transcription rate and chromatin features which are representative of BRG1/EP300 proximate effects (H3 density and nucleosome acetylation), and also looked for cell cycle-related chromatin remodeling (RB1) after short-term MCF7 cell treatment with inhibitors (Figure 4B,C). Loss of BRG1 and EP300 activity immediately reduced gene transcription (Figure 4B) and induced chromatin compaction without a considerable recruitment of RB1 (Figure 4C). In a similar way to long-term deficiency in enzyme activity, decreased acetylation was only found with EP300 inhibition clearly indicates that BRG1 defines chromatin composition dually—by evicting acetylated histones (which ensures transcriptionally permissive and an open chromatin state), and by favoring cell proliferation (precludes assembly of RB1-based repressive complexes). 

To check which of the two described BRG1-dependent modes of chromatin rearrangements is crucial in growth arrested cells, we restored promoter acetylation by treating growth inhibited cells with iHDAC (Figure S2). Reinstatement of nucleosome acetylation (represented by H3K27ac) was insufficient to open chromatin and re-initiate gene transcription in the presence of RB1 that remains associated with gene promoters (Figure 4D). This suggests that cell cycle status overrides the BRG1 activating effect on gene transcription during growth arrest. BRG1-driven expression of proliferation-relevant genes, which potentiates cell divisions, impacts chromatin structure in addition to direct, nucleosome eviction-relevant chromatin opening. Thus, the final chromatin composition at the studied BRG1-driven promoters is defined by cell cycle-dependent and independent aspects, where first corresponds to synthesis of drivers of mitotic divisions, while the latter to acting as a transcriptional co-factor (Figure 4E).

## 3. Discussion

The SWI/SNF chromatin remodeling complex has attracted increasing attention, particularly in cancer biology due to mutations, deletions and insertions in BRG1, and in some of the SWI/SNF core subunits (e.g., SNF5, ARID1A) in ~20% of human tumors. Mutations associated with gain and loss of function, as well as fluctuation in the expression of SWI/SNF components, are linked to the occurrence of cancer and its progression in several ways [15,16]. BRG1 was initially considered as a tumor suppressor, based on, for example, premises from mouse model of primary cells, where inactivation of Brg1 and Snf5 leads to an overall decrease in nucleosome occupancy at a large number of promoters, products of which potentiate cell proliferation [17]. However, more recent data provides evidence that overexpression of some SWI/SNF subunits apparently lacking mutations can be seen as an alternative mechanism by which cellular transformation occurs [18]. As for breast cancer, analysis of the genomic data from TCGA database showed <2% mutation frequency in invasive breast carcinomas [19], whereas elevated expression of BRG1 occurred in 35% to nearly 100% of analyzed primary tumors and is responsible for the high proliferation rate; it also served as a predictive marker for patients at high risk of developing metastases [20,21]. A BRG1-dependent increase in cancer cell division was assigned to (a) upregulated expression of enzymes responsible for fatty acid and lipid biosynthesis by BRG1, which binds at the loci encoding these genes; (b) induction of ABC transporter expression, particularly in response to drug treatment; and (c) association with ER and ER-mediated transcriptional activation [18,22]. Our study also shows the genomic aspect that explains BRG1’s role in breast cancer progression, where the activity of this enzyme maintains a high transcription rate of genes encoding direct drivers of cell proliferation that can act at different cellular levels—signaling cascades (*CDK2*, *CDK4*, *CCNB*—cell cycle phase progression), checkpoints (*CHEK2* responses to genome integrity assessment), DNA replication (*PCNA*—DNA clamp and scaffold for DNA polymerase) and many others listed in Table S2. BRG1 was enriched at >2500 acetylated promoters in MDA-MB-231 cells (Figure 1B); thus their functional association goes beyond proliferation and DNA repair, and comprises inter alia cellular metabolism, response to stress, and regulation of transcription from POLR2A (Figure 1C), i.e., all three mentioned aspects related to BRG1’s role in defining the potential of cancer cells to proliferate. Another crucial feature in cancer biology that renders these cells resistant to anticancer treatment includes the abovementioned presence of ABC transporters in the membrane and DNA repair enzymes in the cytoplasm/nucleoplasm. The latter genes have been documented by us in the current paper to be transcriptionally controlled by BRG1/EP300 complexes in the breast tumors. Thus, a deficiency in BRG1 activity may make cancer cells vulnerable to drugs in two ways, by inhibiting drug efflux, and impairing the removal of DNA damage. An attempt based on BRG1 inhibition might be of particular importance in the treatment of double- or triple-negative breast cancer characterized by the absence of the estrogen receptor (ER), the progesterone receptor (PR) and low to normal levels of HER2, notably where therapeutic approaches targeting these proteins is impossible and patients can benefit only from less specifically targeted cytotoxic drugs. BRG1 functional co-operation with some nuclear receptors were already documented in relatively old reports. Human BRM/hSNF2 alpha and BRG-1/hSNF2 beta, counterparts of yeast SWI2/SNF2 and the *Drosophila* brahma, were identified as activators of estrogen and the retinoic acid receptors at the gene promoters which respond to nuclear receptors [23]. A later mechanistic study revealed that ER targets BRG-1 to the promoters of estrogen-responsive genes in a manner that occurs simultaneously to histone acetylation [24]. BRG1-mediated structural remodeling of chromatin that led to hormone-dependent transcriptional activation by nuclear receptors, was shown to operate in a collaborative manner with histone acetyltransferases such as CREB-binding protein (CBP), p300, and PCAF; this result agrees with our model of co-operation between BRG1 and EP300. These findings also pay attention to the possible use of BRG1 inhibitors or antagonists (once discovered) in the treatment of ER positive cancers in future. In another study, BRG1 suppressed prostate specific antigen (*PSA*) that is transcriptionally controlled by androgen receptor [25]. However, in that model, BRG1-dependent gene repression occurred under the androgen antagonists’-induced growth arrest, where prohibitin recruited BRG1 to PSA promoter and caused eviction of EP300. Also, our study shows that enrichment of BRG1 at some gene promoters was associated with displacement of EP300 from chromatin in response to inhibition of cell proliferation with iCDK4/6, iEP300 and iBRG1. Thus, BRG1 impact on *PSA* expression should be also checked in dividing cells. BRG1 may act as gene activator or suppressor at the target gene promoter depending on the cell cycle status, which can possibly switch the BRG1 role from extrusion of acetylated nucleosome to insertion of their deacetylated form. Again, the molecular trigger (e.g., co-factor, mutual interdependence with BRM, posttranslational modification, element upstream of specific proximal promoter [26]) that can change the influence of the SWI/SNF complex on target gene activation remains unknown. 

In the context of possible use of BRG1 inhibitors in anticancer therapies, of particular importance is our finding regarding BRG1’s co-operation with EP300, since only one non-specific inhibitor of BRG1 is commercially available for scientific purposes. Due to a similar degree of repression of BRG1-dependent genes by EP300 inhibition (Figure 2A–D), such an option extends the possibility of gene targeting by a wider range of compounds. Notably, BRG1 (and perhaps EP300) inhibition might be considered as a therapeutic strategy in other cancer types because BRG1 is overexpressed in other tumor types, although limited insights into how different SWI/SNF subunits drive the development of tumors and complex nature of contribution to defining specific oncogenic pathways clearly requires further investigation [15]. 

Even at the genomic level, chromatin features or DNA sequence that guide BRG1 distribution and association with particular gene promoters or regulatory regions remain poorly defined. It is well acknowledged that this enzyme marks actively transcribed genes and correlates with nucleosome acetylation and trimethylation of H3K4me3, but spatiotemporal mutual interdependence between BRG1’s occurrence and covalent modifications of nucleosomes are also unknown. Since in our study’s SWI/SNF complexes turned out to be readers of nucleosome acetylation governed by EP300-HDAC1 balance intwo distinct cell types (human macrophages and two breast cancer cell lines), one might think that BRG1 confers active gene transcription in other cancer types characterized by BRG1 overexpression. 

Apart from the documented BRG1 role at gene promoters, cancer cell divisions that are transcriptionally controlled by this enzyme regulate expression of numerous functionally-related genes, which may set the phenotype of breast cancer cells. Their link with proliferation is primarily through the presence of the E2F motif, which responds to cell cycle status by recruiting or releasing retinoblastoma-based complexes. However, only ~60% of BRG1/H3K27ac/CpG/E2F-positive genes are overexpressed in fast dividing cells (Figure 1E), suggesting that promoter acetylation in proliferating cells is insufficient to activate transcription, and that unidentified co-factors of BRG1 must operate on or upstream of chromatin. In addition to promoting transcription of genes that potentiate mitotic division, BRG1 has been documented to regulate cell proliferation by co-operating with p53. This tumor suppressor, which displays growth and transformation inhibition functions, adds another link to controlling some of E2F promoters by BRG1. hSNF5 or BRG1 inhibit p53-mediated cell growth arrest and apoptosis by enhancing p53 binding to the promoter of p21—cyclin-dependent kinase inhibitor [27,28]. Such a mode of BRG1 contribution to p53-mediated cell cycle control was observed in MCF7 cells treated with doxorubicin. Recently, BRG1 was found as an activator of p53 transcription in mice embryo-derived P19 cells [29]. These findings make the entire picture of BRG1-proliferation-transcription of cell cycle-controlled genes even more complex, since it depicts BRG1 as a pathway-dependent player. In mitotic dividing cells it promotes proliferation and expression of proliferation-dependent genes, whereas upon cellular stress and p53 activation it contributes to cell growth arrest, where it was shown to repress i.a. nuclear receptor-induced gene transcription [25]. 

As long as the abundance of DNA repair enzymes might not be of particular importance or even antagonize cancer development, the described functional dependence of cancer cell growth on transcription of enzymes capable of removing DNA lesion might be of key importance, not only for cell protection from anticancer therapeutics, but for the maintenance of cancer cell viability and metastases since production of proliferation-associated reactive oxygen species challenges genome integrity and might limit tumor progression. Therefore, BRG1 turns out to be considerable regulator of breast cancer cell physiology. Malignancy-relevant cell cycle re-entrance from a differentiated cell state may globally activate the desired gene expression in a unified mode due to the placement of BRG1-EP300-HDAC1 complexes at promoters that have CpG islands (>85% of BRG1/H3K27ac peaks are associated with CGIs; Figure 1B). However, such a hypothesis requires further experimental confirmation. CpG islands were thought to indicate SWI/SNF independence attributed to the assembly of CpG islands into unstable nucleosomes, but occurrence of BRG1 at the such promoters strongly suggests the possible function of this enzyme in transcription regulation of genes driven by CpG promoters. However, the molecular mechanisms that regulate transcription initiation and elongation from the paused POLR2A pre-initiation complex has not been disclosed [30].

Our study shows that BRG1 makes use of BRG1-activated proliferation promoting gene products and shifts chromatin composition towards a transcriptionally permissive state by allowing nucleosome acetylation. Modified nucleosomes can be further displaced by promoter-bound BRG1. Although BRG1 was found at repressed gene promoters together with RB1, HDAC1, and PRC2, its presence may allow immediate activation of gene transcription upon cell cycle re-entrance [7,31]. Such an idea has been postulated in the past, where BRG1’s association with the promoter of osteocalcin was considered to be a mechanism that ensures re-activation of gene transcription after removal of the proliferation inhibiting factor(s) [32]. However, that paper and others lack information on any mechanistic link between particular chromatin-bound components. Our study explains at least some BRG1-relevant controversies regarding its occurrence, especially at proliferation-driven gene promoters. In dividing cells, BRG1 occurs at the E2F motifs of some cell cycle-dependent genes and, by potentiating expression of genes that promote mitotic cell divisions, BRG1 prevents silencing of their transcription. 

## 4. Materials and Methods

### 4.1. Materials

MCF7 and MDA-MB-231 cell lines were purchased from ATCC and Sigma Aldrich, respectively. DMEM High Glucose w/L-Glutamine w/Sodium Pyruvate, fetal bovine serum and antibiotics (penicillin and streptomycin) were from Biowest (CytoGen, Zgierz, Poland), L15 Medium, iEP300 (C646), iSWI/SNF (PFI-3), iCDK4/6 (PD0332991, palbociclib, Imbrance); anti-rabbit IgG (A0545) and anti-mouse IgG (A4416) (whole molecule)–peroxidase antibody produced in goat, BLUeye prestained protein ladder (#94964), oligonucleotides for real-time PCR were from Sigma Aldrich (Poznan, Poland). Anti-HDAC1 (PA1-860), anti-EP300 (PA1848), Lipofectamine RNAiMAX, OptiMem, Dynabeads™ Protein G, High-Capacity cDNA Reverse Transcription Kit, Click-iT™ Nascent RNA Capture Kit for gene expression analysis, SuperSignal™ West Pico Chemiluminescent Substrate, TRI Reagent™, Silencer™ Select Pre-Designed siRNA ID:s13141 (BRG1), RNase A were from Thermofisher Scientific (Thermofisher Scientific, Warsaw, Poland), while iCDK4/6 (PD0332991) was from Cayman Chemical (Biokom, Janki/Warsaw, Poland).

KAPA HiFi™ HotStart ReadyMix (2X) from KapaBiosystems and Takyon™No ROX SYBR Core Kit blue dTTP from Eurogentec were purchased from Polgen (Łódź, Poland). EvaGreen^®^ Dye, 20X in water was purchased from Biotium (Corporate Place, Hayward, CA, USA). WB antibodies: anti-BRG1 (sc-17796), anti-DNA Ligase I (sc-271678), anti-CDK4 (sc-23896), anti-NEIL3 (sc-393703), anti-PCNA (sc-56), anti-Ki-67 (sc-23900), and anti-cyclin B (CCNB; sc-166210) were purchased from were Santa Cruz Biotechnology (AMX, Lodz, Poland). ChIP grade antibodies: normal rabbit IgG (#2729), BRG1 (#49360), H3K27ac (#4353), anti-histone H3 (#4620), anti-RB1 (#9313), anti-H3K27me3 (#9733), anti-EZH2 (#5246) were purchased from Cell Signaling Technology (LabJOT, Warsaw, Poland).

### 4.2. Cell Culture and Treatment with Inhibitors

MCF7 were cultured in DMEM supplemented with 10% FBS, penicillin/streptomycin (50 U/mL and 50 µg/mL, respectively) in 5% CO_2_, whereas MDA-MB-231 in F15 medium supplemented with 15% FBS, penicillin/streptomycin (50 U/mL and 50 µg/mL, respectively) without CO_2_ equilibration. Both cell lines were maintained in a logarithmic growth phase in a culture and prior to the treatment with any compound. iEP300 (10 µM; C646), iSWI/SNF (10 µM; PFI-3) and iCDK4/6 (1 µM; PD0332991) were added to cells 48 h prior to analysis. Concentration of the studied inhibitors and the time required to induce enzyme inhibition were chosen based on our and other reports. iEP300 and iCDK4/6 at the doses higher than tested induced dramatic cell death in response to longer incubation periods. 

### 4.3. Quantification of Gene Expression

For mRNA quantification the total RNA was extracted with TRI Reagent™, reverse transcribed with High-Capacity cDNA Reverse Transcription Kit and selected cDNA fragments were amplified in real-time PCR (Takyon, Eurogentec; CFX96 C1000 Touch, BioRad Warsaw, Poland) using the following primer pairs: *CDK2*, 5′-CAGGATGTGACCAAGCCAGT-3′ (forward) and 5′-TGAGTCCAAATAGCCCAAGG-3′ (reverse); CDK4, 5′-CTGGTGTTTGAGCATGTAGACC-3′ (forward) and 5′-AAACTGGCGCATCAGATCCTT-3′ (reverse), XRCC2, 5′-TCGCCTGGTTCTTTTTGCA-3′ (forward) and 5′-TCTGATGAGCTCGAGGCTTTC-3′ (reverse), BRCA2, 5′-CTTGCCCCTTTCGTCTATTTG-3′ (forward) and 5′-TACGGCCCTGAAGTACAGTCT-3′ (reverse), LIG1, 5′-CAGAGGGCGAGTTTGTCTTC-3′ (forward) and 5′-AGCCAGTTGTGCGATCTCTT-3′ (reverse), EXO1, 5′-AAACCTGAATGTGGCCGTGT-3′ (forward) and 5′-CCTCATTCCCAAACAGGGACT-3′ (reverse), NEIL3, 5′-GGTCTCCACCCAGCTGTTAAAG-3′ (forward) and 5′-CACGTATCATTTTCATGAGGTGATG-3′ (reverse), PCNA, 5′-TCTGAGGGCTTCGACACCTA-3′ (forward) and 5′-TTCTCCTGGTTTGGTGCTTCA-3′ (reverse); CHEK2, 5′-CAGGTTCTAGCCCAGCCTTC-3′ (forward) and 5′-ACGGAGTTCACAACACAGCA-3′ (reverse); CCNB1, 5′-TGGAGAGGTTGATGTCGAGC-3′ (forward) and 5′-AAGCAAAAAGCTCCTGCTGC-3′ (reverse); BRG1, 5′-AAGAAGACTGAGCCCCGACATTC-3′ (forward) and 5′-CCGTTACTGCTAAGGCCTATGC-3′ (reverse), BRCA1, 5′-TGCCCACAGATCAACTGGAA-3′ (forward) and 5′-CACAGGTGCCTCACACATCT-3′ (reverse); XRCC1, 5′-CGGCGGAAACTCATCCGATA-3′ (forward) and 5′-CCATCAGGGCCTCCTCAAAG-3′ (reverse); ACTB, 5′-TGGCACCCAGCACAATGAA-3′ (forward) and 5′-CTAAGTCATAGTCCGCCTAGAAGCA-3′ (reverse). GAPDH and B2M were from Human Toll-like Receptor Signaling Primer Library (HTLR-I). ACTB, GAPDH, and B2M (HSKG) were used for normalization. Data in figures are shown as Log2FC with respect to untreated control. 

Nascent *CDK4*, *NEIL3*, and *LIG1* mRNA were measured 8 h after cell treatment with iEP300 and iSWI/SNF using Click-iT^®^ Nascent RNA Capture Kit as described previously and was normalized to *ACTB* [33]. 

For protein detection cell lysates were separated with SDS-PAGE, transferred to nitrocellulose membranes and stained overnight with primary antibodies (1.5:10,000) at 4 °C. After staining with HRP-conjugated secondary antibodies (1:10,000; room temperature; 2 h), the signal was developed using SuperSignal™ West Pico Chemiluminescent Substrate and acquired with ChemiDoc-IT2 (UVP, Meranco, Poznan, Poland).

### 4.4. Evaluation of Cell Proliferation

Cell cycle progression in MCF7 and MDA-MB-231 cells treated with iCDK4/6, iEP300 and iSWI/SNF for 48 h was analyzed by flow cytometry as described previously [3]. Additionally, protein level of Ki67, PCNA and CCNB was monitored in cell lysates by western blot. 

### 4.5. Chromatin Immunoprecipitation 

Chromatin immunoprecipitation was carried out according to the protocol previously described [12,19]. Fragments spanning BRG1/H3K27ac/E2F/CpG sites in selected gene promoters were amplified using KAPA HiFi™ HotStart ReadyMix supplemented with EvaGreen^®^ Dye and 4% DMSO and the following primers: *CDK4* prom, 5′-ATAACCAGCTCGCGAAACGA-3′ and 5′-AGAGCAATGTCAAGCGGTCA-3′, *LIG1* prom, 5′-AACACACTCAGATCCGCCAG-3′ and 5′-GCTTCCACCGATTCCTCCTC-3′, *NEIL3* prom, 5′-GTAGGGAGCGACCTCAACAG-3′ and 5′-AGTACAGCCTGGTCCTTCCA-3′.

### 4.6. Transient Gene Silencing

For BRG1 silencing MCF7 and MCD-MB-23 were seeded at the density of 100,000 cells per well and transfected on the following day with RNAiMAX-siRNA complexes prepared in OptiMem according to the following ratio: 20 nmol siRNA and 3 µL of transfection reagent. BRG1 silencing versus non-template control siRNA was confirmed by real-time PCR and western blot 48 h after cell transfection. 

### 4.7. ChIP-Seq Analysis in Galaxy Version 19.05.dev

Publically available, generated by other groups and deposited in the PubMed Central Database data from MDA-MB-231 cells were taken for ChIP-Seq analysis in Galaxy Version 19.05.dev [34]: BRG1—GSM1856026 (SRR2171350), GSM1856027 (SRR2171351) and GSM1856028 (SRR2171352), H3K27ac—GSM1855991 (SRR2171311) and GSM1855992 (SRR2171312); H3K4me3—GSM1700392 (SRR2044734), H3K4me1—GSM2036932 (SRR3096750 and SRR3096751), H3K27me3—GSM949581 (SRR513994), H3K9ac—GSM1619765 (SRR1820123 and SRR1820124), H3—GSM2531568 (SRR5332805), Input—GSM1964894 (SRR2976843). FASTQ quality formats were unified to sanger formatted with FASTQ Groomer [35]. Reads were aligned to Human Genome version 19 using Map with Bowtie for Illumina and unmapped reads were filtered out [36]. ChIP-seq peaks were called in MACS with P value cutoff for peak detection set at 10^−3^ [14]. 

Co-distribution of BRG1 and selected histone modifications in the whole genome was studied by MulitBamSummary/plotCorrelation [37]. In brief, bam files with mapped reads for BRG1 and all studied histone modifications were taken as samples and the genome coverage was computed for equally sized bins (bin size in bp = 1000). For the heatmap in Figure 1A matrix file was generated from the multiBamSummary tool and Pearson correlation was calculated. 

Gene promoters enriched in BRG1, H3K27ac, CpG islands, and E2F were identified by returning intersects of recalled peaks and genomic regions ±2000 bp centered on TSS (overlapping intervals of both datasets) [38]. Genomic intervals for E2F1, E2F4, and CpG Islands were taken from UCSC Main tables wgEncodeRegTfbsClusteredV3 and cpgIslandExt, respectively. Venn diagrams were created in http://www.interactivenn.net/ from gene lists. Annotation of differentially expressed genes to gene ontology terms was carried out in GOrilla (using two unranked lists of genes and complete list of genes expressed in MDA-MB-231 as a background). 

The following data from normal breast, primary tumor, MCF7 and MDA-MB-231 cells were taken for RNA-Seq analysis: normal breast—GSM1695870 (SRR2040339), GSM1695872 (SRR2040341), GSM1695873 (SRR2040342), GSM1695874 (SRR2040343), GSM1695877 (SRR2040346), GSM1695878 (SRR2040347); primary tumor—GSM1695891 (SRR2040360), GSM1695898 (SRR2040367), GSM1695899 (SRR2040368), GSM1695882 (SRR2040351), GSM1695890 (SRR2040359), GSM1695894 (SRR2040363); MCF7—GSM2422725 (SRR5094305), GSM2422726 (SRR5094306), GSM2422727 (SRR5094307), GSM2422728 (SRR5094308), GSM2422729 (SRR5094309), GSM2422730 (SRR5094310); MDA-MB-231 - GSM2422731 (SRR5094311), GSM2422732 (SRR5094312), GSM2422733 (SRR5094313), GSM2422734 (SRR5094314), GSM2422735 (SRR5094315), GSM2422736 (SRR5094316).

Having FASTQ quality formats unified to sanger formatted with FASTQ Groomer reads were mapped to Human Genome version 19 using TopHat [39]. Transcripts were assembled with Cufflinks (using UCSC Known Gene as a reference annotation) and merged with Cuffmerge [40]. Differential gene expression in cancer versus normal breast cells was calculated with Cuffdiff and shown as heatmap for two selected GOs (mitotic cell cycle process and DNA repair). Frequencies of transcription factors and chromatin remodelers occurrence at the promoters of genes that were overexpressed in all three cancer cell types (Log2FC > 0.5) were scored based on results of bedtools Intersect intervals (gene promoters spanning 2 kbp from TSS and UCSC Main tables wgEncodeRegTfbsClusteredV3 and tfbsConsSites) [38].

### 4.8. Statistical Analysis

Data in Table S3 are shown as mean ± standard deviation of the mean (SEM). Student’s *t*-test was used to determine statistically significant differences between two means (marked with * when *p*  <  0.05, ** when *p* < 0.01, *** when *p* < 0.001), while one-way analysis of variance (ANOVA) was carried out in GraphPad Prism 5 to compare means in several groups (marked with * when *p* < 0.05, ** when *p* < 0.01, *** when *p* < 0.001). 

## 5. Conclusions

Summarizing, activity of one chromatin-associated enzyme defines the expression profile of numerous genes in breast cancer cells by acting directly on chromatin, and by promoting cell cycle progression. BRG1 removes acetylated nucleosomes, thereby facilitating transcription and preventing recruitment of retinoblastoma-based repressive complexes to E2F-driven promoters. Thus, BRG1 defines key breast cancer features in the cell lines we have investigated. Inhibitors of BRG1 and EP300 can be considered as future anticancer drugs that can arrest cell growth and/or render them sensitive to DNA damaging agents. 

## Supplementary Materials

The following are available online at https://www.mdpi.com/2072-6694/12/2/349/s1, Figure S1: Efficiency of BRG1 silencing; Figure S2: Effect of HDAC inhibitor on gene promoters in G1 arrested cells; Figure S3: Whole western blot images; Table S1: BRG1 and H3K27ac distribution at the E2F/CpG positive gene promoters; Table S2: Gene ontology; Table S3: Statistical analysis.
