# Supplementary material for: BRG1 Activates Proliferation and Transcription of Cell Cycle-Dependent Genes in Breast Cancer Cells
Source: Cancers (Basel). 2020 Feb 4;12(2):349. doi: 10.3390/cancers12020349 (PMC7072512; doi:10.3390/cancers12020349)
Supplement: Supplementary file 1 [file cancers-12-00349-s001.zip › cancers-691070-suppl-final/cancers-691070-supplementary figures/Figure S3.pptx]

## Slide 1
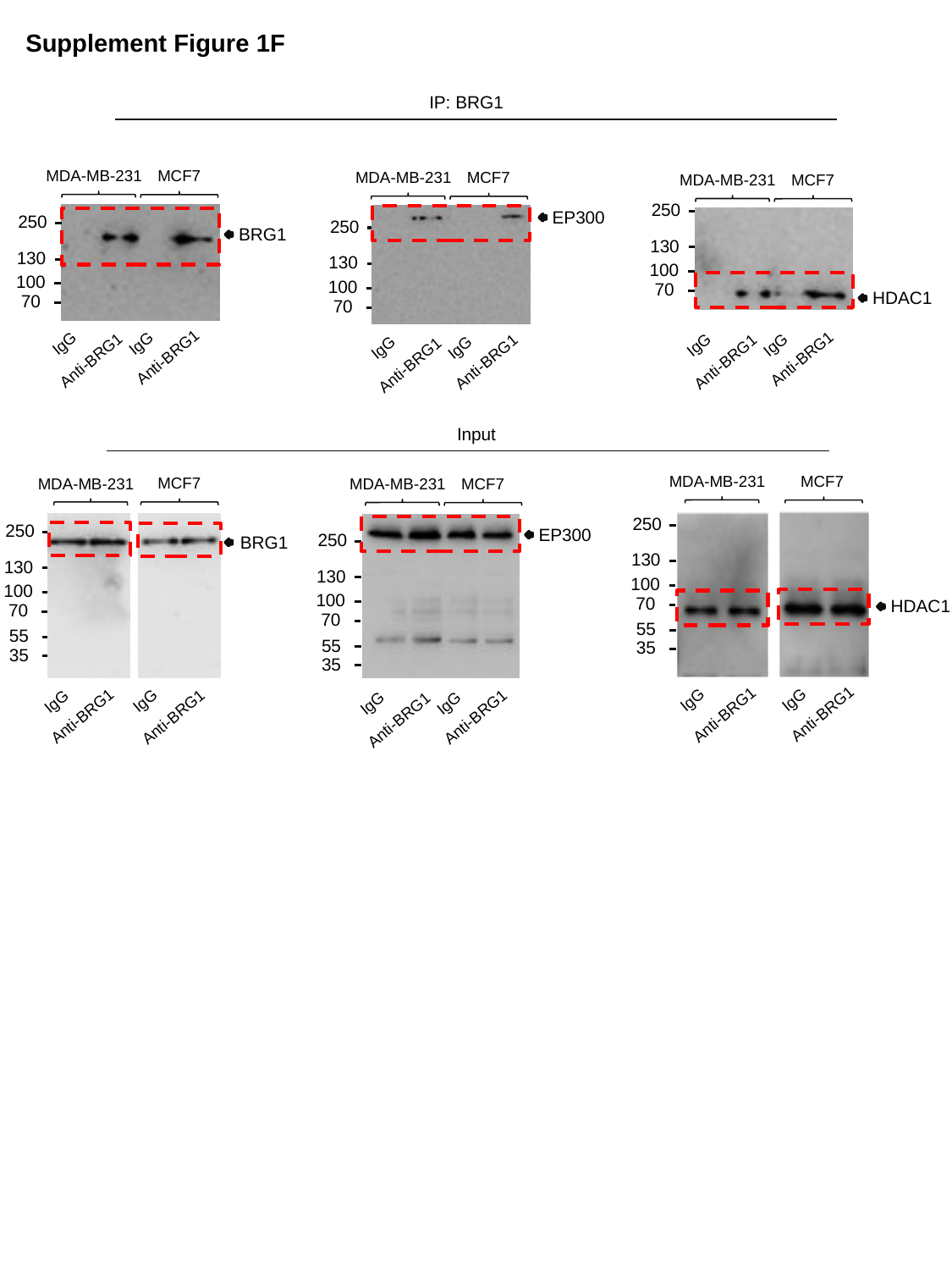

Supplement Figure 1F
IP: BRG1
MCF7
MDA-MB-231
MCF7
MDA-MB-231
MCF7
MDA-MB-231
250
130
100
70
EP300
250
130
100
70
250
130
100
70
BRG1
HDAC1
IgG
IgG
IgG
IgG
IgG
IgG
Anti-BRG1
Anti-BRG1
Anti-BRG1
Anti-BRG1
Anti-BRG1
Anti-BRG1
Input
MCF7
MDA-MB-231
250
130
100
70
55
35
HDAC1
MCF7
MDA-MB-231
MCF7
MDA-MB-231
250
130
100
70
55
35
BRG1
EP300
250
130
100
70
55
35
IgG
IgG
IgG
IgG
IgG
IgG
Anti-BRG1
Anti-BRG1
Anti-BRG1
Anti-BRG1
Anti-BRG1
Anti-BRG1

## Slide 2
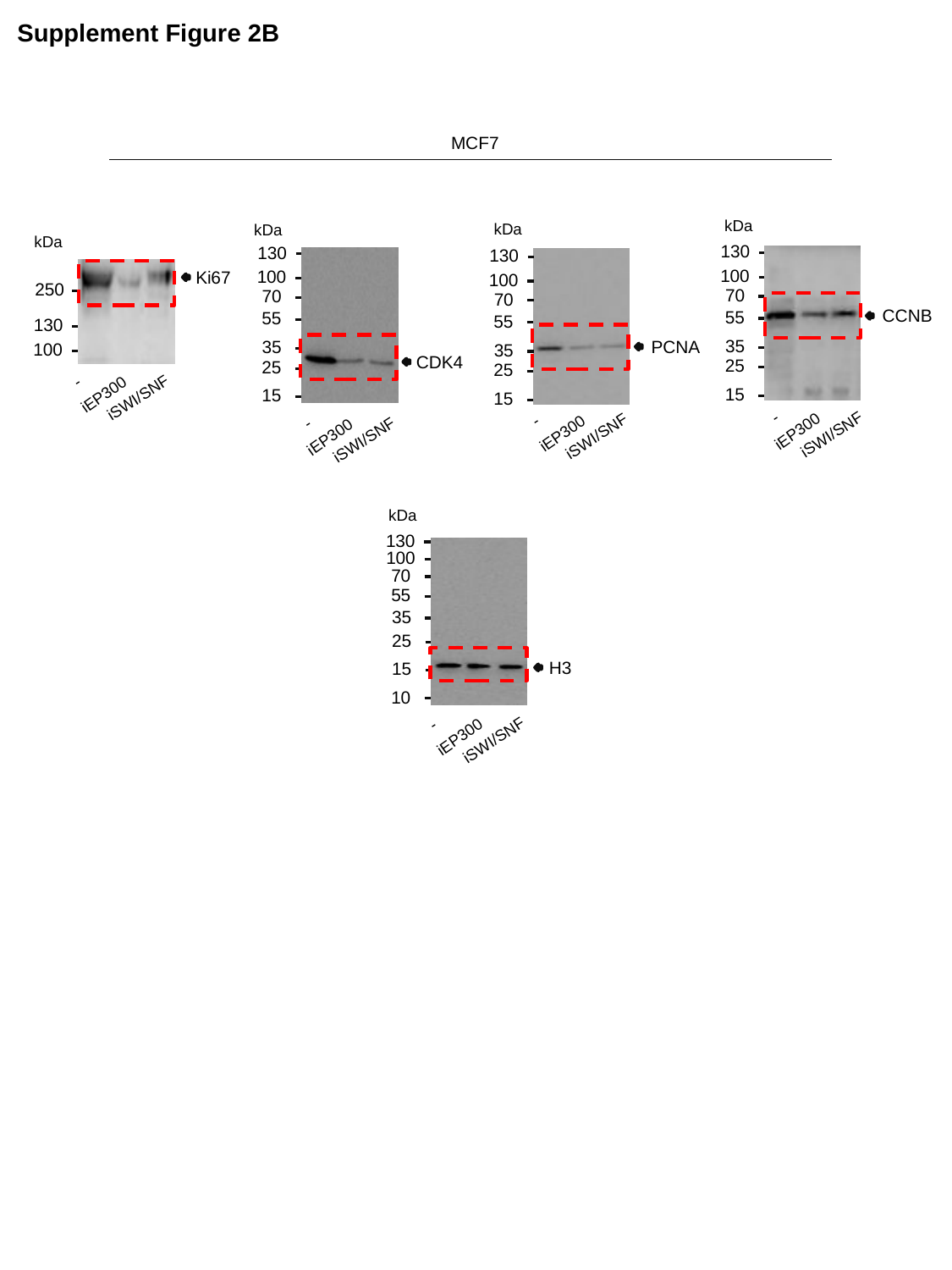

Supplement Figure 2B
MCF7
kDa
130
100
70
55
35
25
15
CCNB
-
iEP300
iSWI/SNF
kDa
130
100
70
55
35
25
15
PCNA
-
iEP300
iSWI/SNF
kDa
130
100
70
55
35
25
15
CDK4
-
iEP300
iSWI/SNF
kDa
Ki67
250
130
100
-
iEP300
iSWI/SNF
kDa
130
100
70
55
35
25
15
10
H3
-
iEP300
iSWI/SNF

## Slide 3
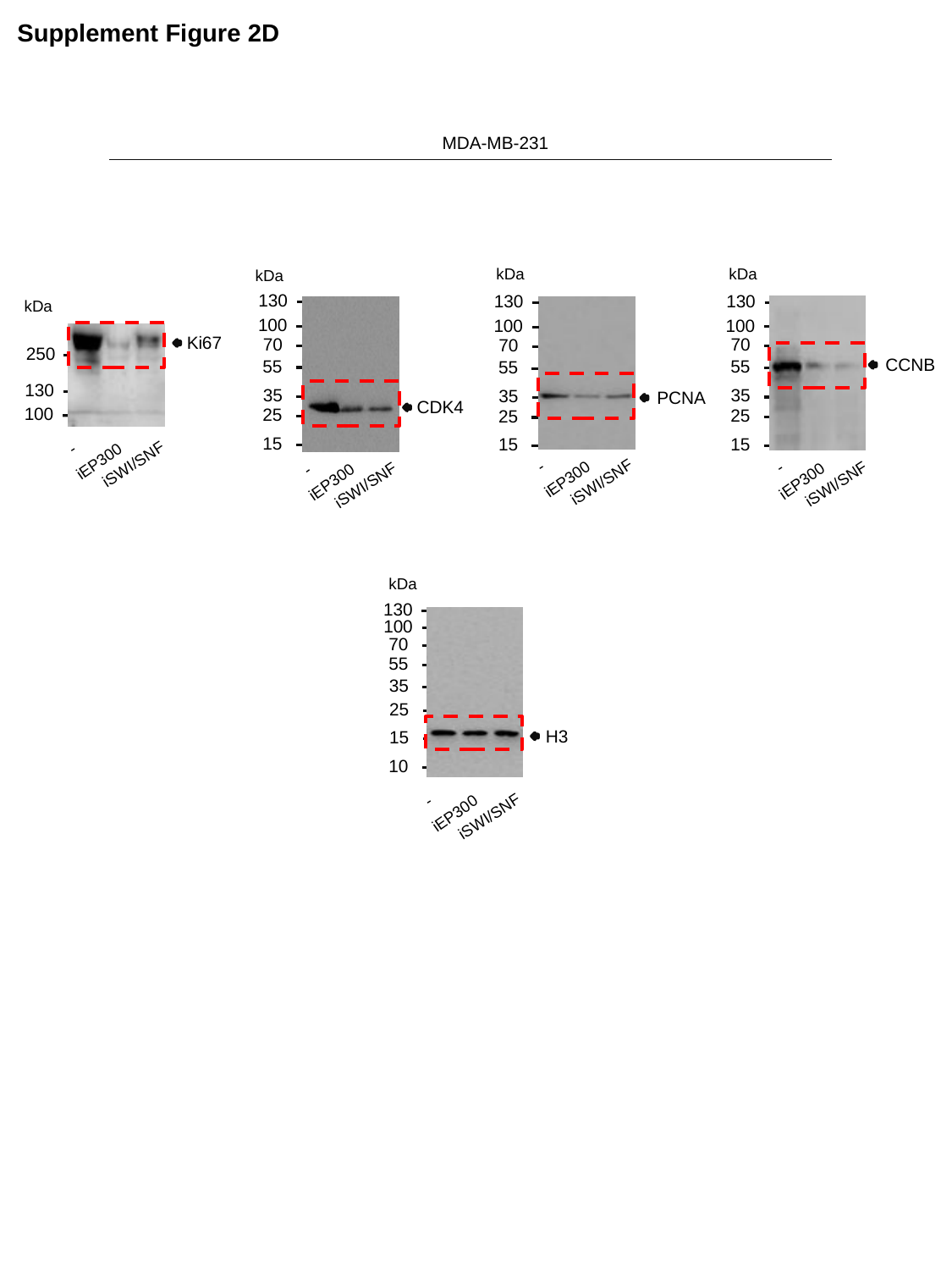

Supplement Figure 2D
MDA-MB-231
kDa
130
100
70
55
35
25
15
PCNA
-
iEP300
iSWI/SNF
kDa
130
100
70
55
35
25
15
CCNB
-
iEP300
iSWI/SNF
kDa
130
100
70
55
35
25
15
CDK4
-
iEP300
iSWI/SNF
kDa
Ki67
250
130
100
-
iEP300
iSWI/SNF
kDa
130
100
70
55
35
25
15
10
H3
-
iEP300
iSWI/SNF

## Slide 4
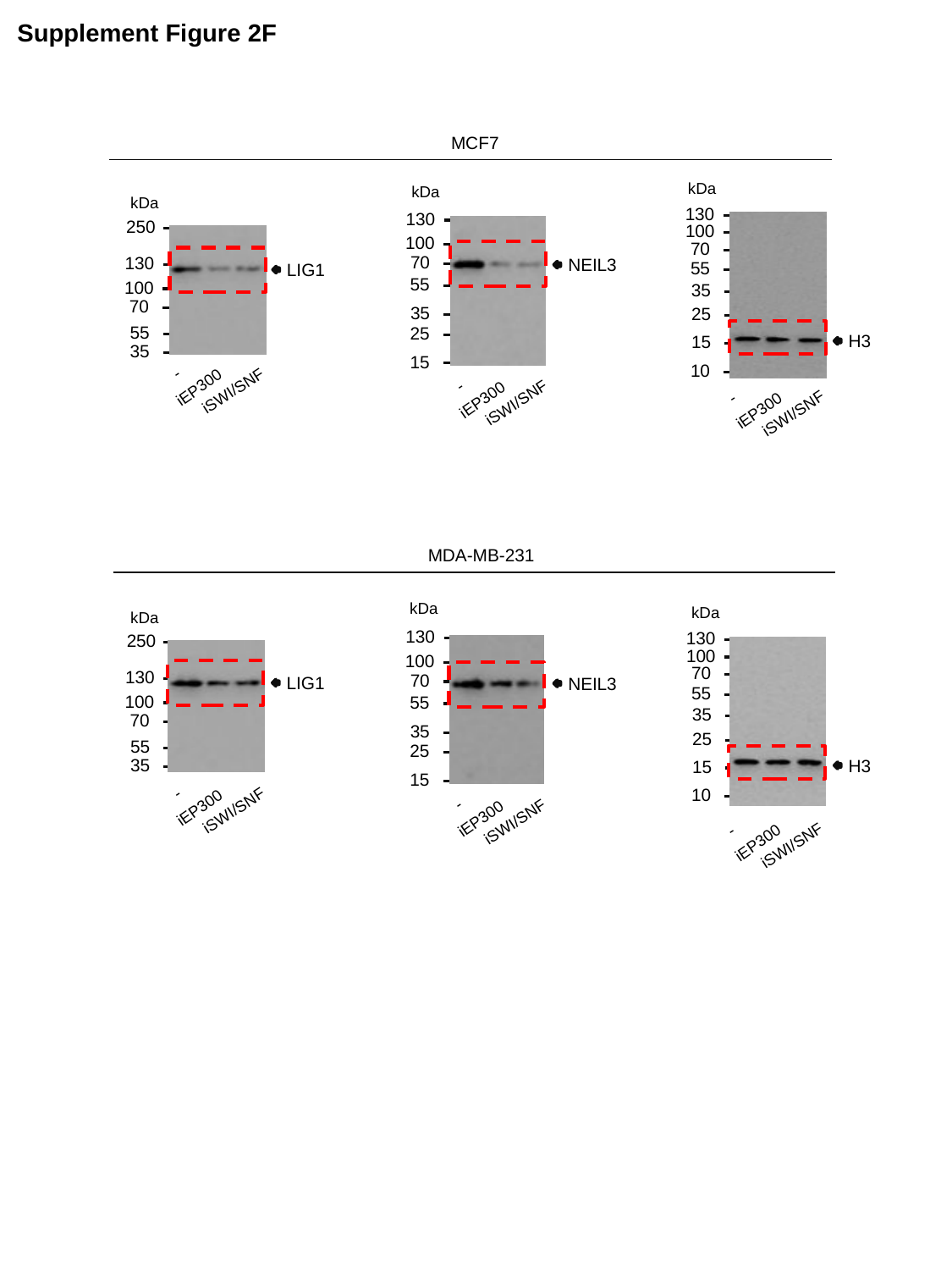

Supplement Figure 2F
MCF7
kDa
130
100
70
55
35
25
15
10
H3
-
iEP300
iSWI/SNF
kDa
130
100
70
55
35
25
15
NEIL3
-
iEP300
iSWI/SNF
kDa
250
130
100
70
55
35
LIG1
-
iEP300
iSWI/SNF
MDA-MB-231
kDa
130
100
70
55
35
25
15
NEIL3
-
iEP300
iSWI/SNF
kDa
130
100
70
55
35
25
15
10
H3
-
iEP300
iSWI/SNF
kDa
250
130
100
70
55
35
LIG1
-
iEP300
iSWI/SNF

## Slide 5
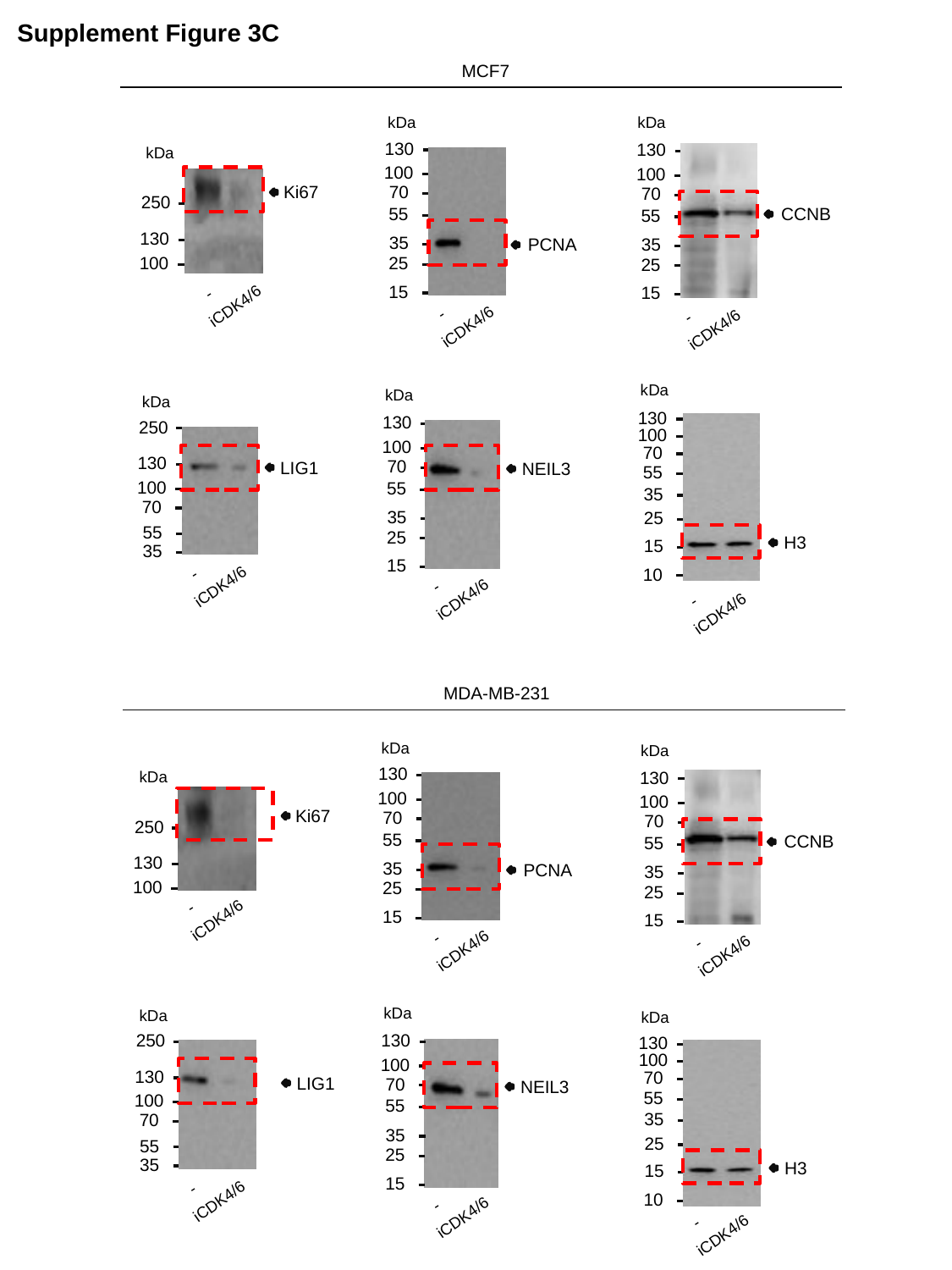

Supplement Figure 3C
MCF7
kDa
130
100
70
55
35
25
15
PCNA
kDa
130
100
70
55
35
25
15
CCNB
kDa
Ki67
250
130
100
-
iCDK4/6
-
iCDK4/6
-
iCDK4/6
kDa
130
100
70
55
35
25
15
10
H3
-
iCDK4/6
kDa
130
100
70
55
35
25
15
NEIL3
-
iCDK4/6
kDa
250
130
100
70
55
35
LIG1
-
iCDK4/6
MDA-MB-231
kDa
130
100
70
55
35
25
15
PCNA
-
iCDK4/6
kDa
130
100
70
55
35
25
15
CCNB
-
iCDK4/6
kDa
Ki67
250
130
100
-
iCDK4/6
kDa
130
100
70
55
35
25
15
NEIL3
-
iCDK4/6
kDa
250
130
100
70
55
35
LIG1
-
iCDK4/6
kDa
130
100
70
55
35
25
15
10
H3
-
iCDK4/6
