# Supplementary figures and images for: BRG1 Activates Proliferation and Transcription of Cell Cycle-Dependent Genes in Breast Cancer Cells
Source: Cancers (Basel). 2020 Feb 4;12(2):349. doi: 10.3390/cancers12020349 (PMC7072512; doi:10.3390/cancers12020349)

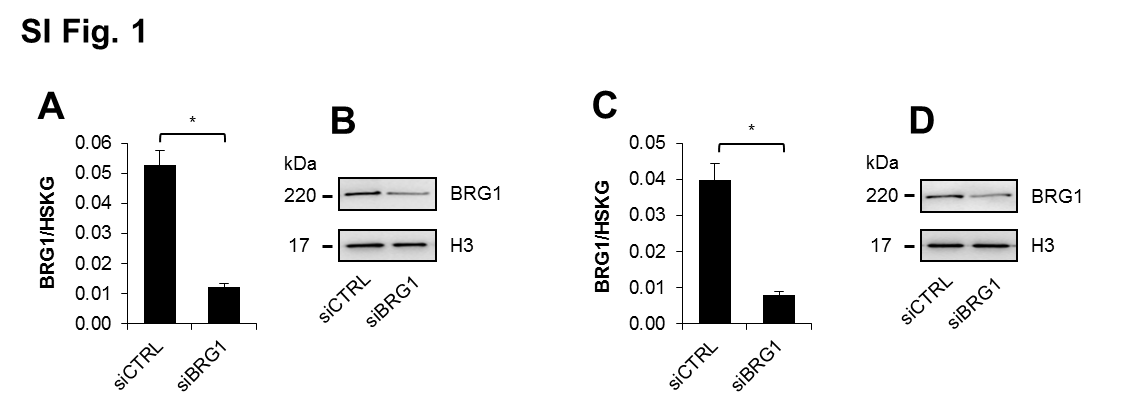

Supplement: Supplementary file 1 [file cancers-12-00349-s001.zip › cancers-691070-suppl-final/cancers-691070-supplementary figures/Figure S1.tif]

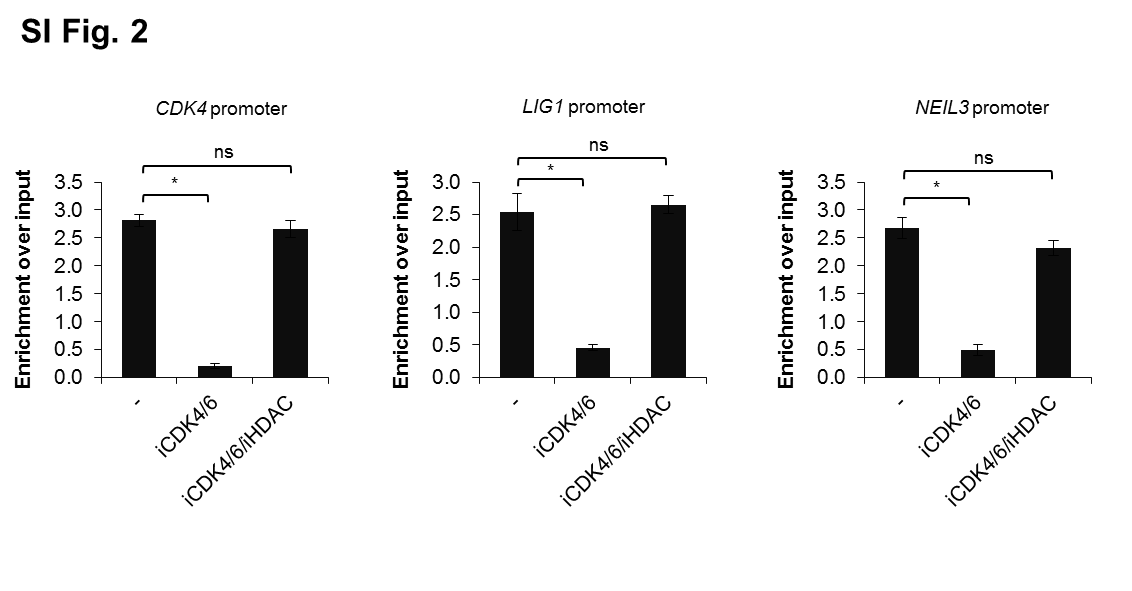

Supplement: Supplementary file 1 [file cancers-12-00349-s001.zip › cancers-691070-suppl-final/cancers-691070-supplementary figures/Figure S2.tif]
